# Comparison of Insomnia, Depression, and Perceived Social Support among Individuals with Amphetamine Use Disorder (AUD) and Healthy Controls

**DOI:** 10.31083/AP38786

**Published:** 2025-02-28

**Authors:** Nasrin Abdoli, Dena Sadeghi-Bahmani, Nader Salari, Mehdi Khodamoradi, Zeno Stanga, Annette B. Brühl, Serge Brand, Kenneth M. Dürsteler

**Affiliations:** ^1^Substance Abuse Prevention Research Center, Health Institute, Kermanshah University of Medical Sciences, 6719851115 Kermanshah, Iran; ^2^Department of Psychology, Stanford University, Stanford, CA 94305, USA; ^3^Department of Epidemiology and Population Health, Stanford University, Stanford, CA 94305, USA; ^4^Department of Biostatistics, School of Health, Kermanshah University of Medical Sciences, 6719851115 Kermanshah, Iran; ^5^Sleep Disorders Research Center, Kermanshah University of Medical Sciences, 6719851115 Kermanshah, Iran; ^6^Division of Diabetes, Endocrinology, Nutritional Medicine and Metabolism, University Hospital and University of Berne, 3010 Berne, Switzerland; ^7^Centre of Competence for Military and Disaster Medicine, Swiss Armed Forces, 3008 Berne, Switzerland; ^8^Center for Affective, Stress and Sleep Disturbances, Psychiatric Clinics of the University of Basel, 4002 Basel, Switzerland; ^9^Faculty of Medicine, University of Basel, 4052 Basel, Switzerland; ^10^School of Medicine, Tehran University of Medical Sciences, 1417466191 Tehran, Iran; ^11^Center for Disaster Psychiatry and Disaster Psychology, Psychiatric Clinics of the University of Basel, 4002 Basel, Switzerland; ^12^Division of Substance Use Disorders, Psychiatric University Clinics Basel, 4002 Basel, Switzerland; ^13^Department for Psychiatry, Psychotherapy and Psychosomatic, Psychiatric Hospital, University of Zurich, 8001 Zurich, Switzerland

**Keywords:** amphetamine use disorder, depression, insomnia, social support

## Abstract

**Background:**

Compared to the general population, individuals with substance use disorders (SUD) report more frequently to suffer from sleep disturbances and symptoms of depression, and to perceive lower social support. Here, we investigated whether this pattern of mental health issues could be confirmed and replicated among individuals with amphetamine use disorder (AUD). We also assessed the degree of perceived social support from their families, friends and significant others, always compared to healthy controls (HC) of the general population.

**Method:**

Individuals with AUD attending the Outpatient Department for Substance Abuse of the Kermanshah University of Medical Sciences (Kermanshah, Iran) (n = 468; 30.8% females; mean age: 29.16 years) and healthy controls (HC; n = 376; 34.6% females; mean age: 24.11 years) participated in the study. Participants completed a series of self-rating questionnaires covering sociodemographic information, symptoms of insomnia and depression, and perceived social support from their families, friends and significant others.

**Results:**

Compared to HC, individuals with AUD reported higher scores for insomnia and depression, and lower scores for perceived social support (families; friends; significant others). Older age and higher severity scores for depression and insomnia were the predictors in the binary logistic regression model to identify individuals with AUD and HC with a precision of 97.4%.

**Conclusions:**

Individuals with AUD additionally suffer from insomnia and depression, along with lower perceived social support. Given that standardized intervention programs for insomnia, depression and social competencies exist, such interventions might mitigate mental health issues among individuals with AUD and improve their psychosocial behavior.

## Main Points

∙ We compared individuals with amphetamine use disorder (AUD) and healthy controls (HC).

∙ Compared to HC, individuals with AUD reported higher scores for insomnia and depression.

∙ Compared to HC, individuals with AUD reported lower scores for perceived social support by their families, friends and significant others.

∙ Older age and higher scores for depression and insomnia predicted individuals with AUD and HC.

## 1. Introduction

Drug use is one of the globally most demanding health issues, trialing a person’s mental health, a person’s social environment, along with the national health system and the public domain [1, 2]. The latest World Drug Report reporting data from 2021 showed that about 457 million people use illicit drugs [2].

Amphetamines, after cannabis and opioids, are the third most commonly used illicit substances worldwide [2]. Amphetamine is a central nervous system (CNS) stimulant used for the treatment of attention-deficit/hyperactivity disorder (ADHD) [3, 4, 5] and narcolepsy [6, 7]. At therapeutic dosages, amphetamine impacts on emotion (e.g., increased mood), cognition (e.g., increased and sustained attention and vigilance; cognitive control), and behavior (e.g., shorter reaction time, fatigue resistance, and increased muscle strength; see [8] for a more thorough overview). For non-medical purposes, amphetamine is used as a club drug at much higher and thus non-therapeutic dosages for its energetic and euphoric effect. In addition, amphetamine is often combined with other substances such as alcohol and downers (i.e., sedatives, hypnotics and narcotics). Individuals taking amphetamine for non-medical use report increased stimulation, alertness, prolonged wakefulness, and more intense perception of external stimuli. On the flip side, adverse effects can be serious and life-threatening. To name just but a few, cardiovascular issues (hypertension, hypotension or tachycardia [9]), gastrointestinal side-effects, appetite loss, dry mouth, excessive grinding of the teeth, nosebleed, tics and weight loss [10], and, most importantly, amphetamine use disorder (AUD). Amphetamine use disorder was virtually solely observed among non-medical amphetamine users self-administered at high doses, and not, when prescribed at therapeutic dosages for ADHD and narcolepsy [10].

According to data from 2015 as published in the World Drug Report 2023 [2], the highest prevalence rates for substance use in Iran were observed for opioids, followed by amphetamine and cannabis. In the present study, we focused exclusively on individuals with amphetamine use disorder (AUD) undergoing medical and psychotherapeutic treatment in an outpatient emergency center.

### 1.1 Sleep and Substance Use Disorder (SUD) and Amphetamine Use Disorder (AUD)

For sleep, there is extensive evidence of the tight sleep-psychological functioning-link [11, 12, 13, 14]. To illustrate, findings from meta-analyses and systematic reviews showed that insomnia is causally related to the emergence and maintenance of a broad variety of psychiatric disorders [12, 13, 14], and unsurprisingly, treating insomnia impacted favorably on symptoms of depression [15].

For sleep and AUD, compared to former amphetamine users and non-amphetamine users, current amphetamine users reported higher scores for distress and poor subjective sleep, including more sleep disturbances [16]. Further, higher scores for major depressive disorder and distress predicted poor sleep [16]. Methamphetamine (MA) use was associated with the need to extent wakefulness and to reduce sleep need, and to manage wakefulness and sleep need while under MA. Further, sleep disruptions after ceasing the effect of MA was a major trigger to re-use MA [17]. However, no research has focused so far on the association between symptoms of insomnia and depression, in relation to social support among individuals with current AUD, and compared to healthy controls (HC). Given this, the present study aimed to bridge this gap of research.

### 1.2 Depression and Amphetamine Use Disorder (AUD)

For the specific amphetamine-depression-link, it appears that data are missing. For the *meth*amphetamine-depression-link, a recent meta-analysis and systematic review summarized data from 14 studies as follows: Compared to the general population, individuals with *meth*amphetamine use disorder had a 2.80-higher risk to suffer from symptoms of depression [18]. Thus, the gap of research is apparent: No study has focused so far on symptoms of depression among individuals with AUD, and no research related such symptoms of depression to insomnia and social support, always among individuals with AUD, and compared to healthy controls (HC).

### 1.3 Loneliness, Social Support and Substance Use Disorders (SUD)

As regards social support or loneliness, that is, the lack of subjectively perceived support, several lines of research found that individuals with SUD reported higher scores for loneliness, compared to the general population [19]. Among 594 participants aged 14 to 24 years, Bonar *et al*. [19] observed that higher consumption of alcohol and cannabis and negative peer relationships and parental substance use at baseline predicted higher scores for loneliness 24 months later. By contrast, higher parental social support at baseline predicted lower scores for loneliness. The strengths of that study was the longitudinal design, the observation of possible bi-directional influences between substance use, the quality of peer and parental relationships and a person’s loneliness as a proxy of social behavior. Relatedly, Ingram *et al*. [20] summarized in their systematic review covering 41 studies that among individuals with substance use problems, loneliness was associated with poorer physical and mental health, more problematic social relationships, being stigmatized and the perception of ill-being by others. Shahid and Asmat [21] summarized eight studies in their systematic review and concluded that being stigmatized was associated with lower treatment outcome. Importantly, negative comments from close relations were associated with higher rates of relapses. By contrast, perceived social support was associated with a constructive impact on the treatment. Next, individuals with SUD reported lower scores for quality of life, but comparable quality of life scores of other psychiatric disorders [22]. Importantly, higher scores for quality of life were associated with perceived social support from family and friends. As such, a broader and more stable social network seemed to be associated with better social health. Further, the degree of family cohesion and social support was lower among individuals with SUD, when compared to individuals with physical disorders [23]. By contrast, higher family cohesion and higher social support were associated with more favorable treatment outcomes. Among 60 studies covering individuals with SUD aged 18 to 60 years, those individuals remaining abstinent three to six months after SUD treatment were also those reporting a more stable and encouraging social support [24]. Similarly, the degree of social support was associated with more favorable treatment outcomes among individuals with SUD [25, 26, 27, 28]. Last, Winters *et al*. [29] investigated in their systematic review and meta-analysis the associations between an adolescent person’s cognitive-emotional processes of social interactions and SUD, and the authors showed that callous-unemotional traits, understood as a fundamental insecure-avoidant and defiant attachment and interactional style, were associated with higher substance use issues. The beauty of this review was that theories of social cognition, including a person’s cognitive-emotional elaboration of social interactions, were considered when evaluating an adolescent’s SUD.

To our understanding, specific research on social issues in individuals with AUD is missing in general, and in Iran, in specific. We took these observations into consideration and asked, whether and to what extent individuals with AUD reported similar or dissimilar dimensions of social support from their family, peers and significant others, compared to the general population.

### 1.4 The Present Study

Individuals with SUD and AUD reported higher prevalence rates of symptoms of insomnia [16, 30, 31, 32, 33, 34] and depression [18, 35, 36, 37]. By contrast, compared to the general population, individuals with SUD and AUD also reported more social issues in terms of less social support, while social support turned out to be a favorable factor for the treatment of SUD [19, 25, 26, 27, 28, 29].

Given this, and for want of specific data on individuals with AUD in Iran, we built-up the hypotheses based on general observations on individuals with SUD.

The two hypotheses and research questions were: First, following others [16, 18, 30, 31, 32, 33, 34, 35, 36, 37]. we assumed that individuals with AUD would report higher scores for insomnia and depression, compared to healthy controls (HC). Second, following previous research [19, 25, 26, 27, 28, 29], we expected that individuals with AUD compared to HC would report lower scores for social support. With the research question we asked, whether and to what extent symptoms of depression, insomnia and social support from the family, peers and significant others would predict to assign individuals with SUD and HC correctly to the specific study condition. We claim that the present study has the potential to shed some more light on the associations between symptoms of insomnia and depression, along with perceived social support among a ‘pure’ sample of individuals using amphetamines on a regular basis of use disorder (AUD), and compared to healthy controls (HC).

## 2. Methods

### 2.1 Procedure

Individuals with AUD and HC were approached to participate at the present cross-sectional study. All participants were informed about the aims of the study and the anonymous and secure data handling. Afterwards, participants signed the written informed consent, and they completed a series of self-rating questionnaires on sociodemographic information, symptoms of depression and insomnia, and on perceived social support (see details below). The Ethics Committee of Kermanshah University of Medical Sciences, Kermanshah, Iran (ethics code: IR.KUMS.REC.1400.186) approved the study, which was performed in accordance with the current version [38] of the Declaration of Helsinki.

### 2.2 Participants

For individuals with AUD inclusion criteria were: (1) Age 18 years and higher. (2) Male or female at birth. (3) Suffering from AUD, as ascertained by a trained psychiatrist or clinical psychologist of the Outpatient Department for Substance Abuse of the Kermanshah University of Medical Sciences (Kermanshah, Iran). Diagnosis was based on an extensive and thorough clinical interview for DSM-5 disorders [39]. (4) Use of amphetamine for non-medical reasons. (5) Meeting two to five criteria for AUD based on the DSM-5 [40]. (6) Able and willing to comply with the study conditions. (7) Signed written informed consent.

Exclusion criteria were: (1) Another psychiatric disorder was the main diagnosis, such as anti-social personality disorder, major depressive disorder, bipolar disorder, anxiety disorders, PTSD, other SUD such as opioid use disorder, cannabis use disorder, cocaine use disorder, or methamphetamine use disorder. (2) Neurological disorders such as multiple sclerosis, substance-induced movement disorders, or cognitive disorders, as ascertained by an experienced neurologist. (3) Pregnancy or breastfeeding, as they may alter mood and sleep states. Tobacco use was not an exclusion criterion.

Of the 712 individuals approached, 468 (53.84%) participated at the study.

#### Healthy Controls

The study was posted on the intranet of the universities and university hospitals of the Kermanshah University of Medical Sciences (Kermanshah, Iran).

Inclusion criteria were: (1) Age 18 years and higher. (2) Male or female at birth. (3) Able and willing to comply with the study conditions. (4) Signed written informed consent. Exclusion criteria were: (1) Any kind of current psychiatric disorders or neurological disorders, as mentioned above. (2) Pregnancy or breastfeeding, as they may alter mood and sleep states. Tobacco use was not an exclusion criterion.

Of the 390 individuals approached, 376 (96.3%) participated at the study.

### 2.3 Measures

#### 2.3.1 Sociodemographic Information

Participants reported on their age (years), gender at birth (female; male), civil status (single; married; divorced), highest educational degree (under diploma; diploma; high school degree; bachelor’s degree or higher), and physical activity (yes; no).

#### 2.3.2 Insomnia

To assess insomnia, participants completed the Farsi version [41] of the Athens Insomnia Scale [42]. It consists of eight items, focusing on the ICD-10 criteria for insomnia. Answers are given on 4-points Likert scales ranging from 0 (= normal; no problem) to 3 (= very decreased; intense), with higher sum scores reflecting a higher intensity of insomnia. Further, the cut-off of 6 points indicates insomnia (Cronbach’s alpha: 0.82).

#### 2.3.3 Depression

To assess symptoms of depression, participants completed the Farsi version [43] of the Kutcher Adolescent Depression Scale (KADS) [44], which was also validated for adult samples [45]. The questionnaire consists of 11 items. Typical items are: “Over the last week, how have you been “on average” or “usually” regarding the following items: Low mood, sadness, feeling blah or down, depressed, just can’t be bothered.”; or: “Feelings of worthlessness, hopelessness, letting people down, not being a good person.” Answers are given on 4-points Likert scales ranging from 0 (= hardly ever) to 3 (= most of the time), with higher sum scores reflecting a higher severity of symptoms of depression (Cronbach’s alpha: 0.79).

#### 2.3.4 Perceived Social Support

To assess social support, participants completed the Farsi version [46] of the Multidimensional Scale of Perceived Social Support [47]. It consists of 12 items. Typical items are (family): “I can talk about my problems with the family.”; (friends): “I can talk about my problems with my friends.”; (significant other): “There is a special person in my life who cares about my feelings”. Answers are given on 5-point Likert scale ranging from 1 (= strongly disagree) to 5 (= to strongly agree), with higher sum scores reflecting higher support from the family, friends and significant others. Further, an overall sum score is reported (Cronbach’s alpha = 0.89).

### 2.4 Statistical Analysis

A series of χ^2^-tests was performed to compare sociodemographic information between participants with AUD and HC.

A series of *t*-tests was performed to compare insomnia, depression and perceived social support between participants with and without AUD.

With a series of Pearson’s correlations, we calculated the associations between insomnia, depression, and social support (family, friends, significant others).

A binary logistic regression model was finally performed to calculate whether scores for insomnia, depression, and perceived social support could predict participants’ correct assignment to the group with and without AUD.

The level of significance was set at alpha <0.05. All statistical procedures were performed with SPSS® version 29.0 (IBM Corporation, Armonk, NY, USA) for Apple Mac®.

## 3. Results

### 3.1 General Sociodemographic Information

Table 1 reports the descriptive and inferential statistical indices of sociodemographic information (categorical variables) of participants with AUD and HC, while the age comparison is shown in Table 2.

**Table 1.** **Descriptive and inferential statistical comparisons of sociodemographic information (categorical variables) between individuals with AUD and HC**.

Dimensions		AUD	HC	Statistics
		N (%)	N (%)	χ^2^-tests
Gender	Female	144 (30.8)	130 (34.6)	χ^2^ (N = 844, df = 1) = 1.37
	Male	324 (69.2)	246 (65.4)	
	Total	468 (100.0)	376 (100.0)	
Marital status	Single	300 (64.1)	308 (81.9)	χ^2^ (N = 844, df = 2) = 36.35***
	Married	158 (33.8)	68 (18.1)	
	Divorced	10 (2.1)	0 (0)	
	Total	468 (100.0)	376 (100.0)	
Education	<Diploma	144 (30.8)	186 (49.5)	χ^2^ (N = 844, df = 3) = 48.75***
	Diploma	130 (27.8)	114 (30.3)	
	>Diploma	90 (19.2)	38 (10.1)	
	Bachelor and more	104 (22.2)	38 (10.1)	
	Total	468 (100.0)	376 (100.0)	
Occupational status	Students	163	242	χ^2^ (N = 844, df = 3) = 154.99***
	Unemployed	153	39	
	Independent worker	28	38	
	Employed	124	25	

Notes: AUD, amphetamine use disorder; HC, healthy controls; *** = *p* < 0.001.

**Table 2.** **Descriptive and inferential statistical comparisons of age, insomnia, depression and social support between participants with AUD and HC**.

		Group		Statistics
		AUD	HC	
		M (SD)	M (SD)	
Age (years)		29.16 (6.62)	24.11 (5.23)	*t*(842) = 1.23, *p* > 0.1, d = 0.23 [S]
Insomnia		19.90 (1.88)	10.80 (3.24)	*t*(842) = 50.85, *p* < 0.001, d = 3.5 [L]
Depression		28.46 (4.28)	22.48 (3.37)	*t*(842) = 22.09, *p* < 0.001, d = 1.52 [L]
Social support				
	Family	18.05 (5.99)	16.81 (6.19)	*t*(842) = 5.90, *p* < 0.001, d = 0.41 [S]
	Friends	12.77 (4.70)	13.79 (4.70)	*t*(842) = 3.11, *p* < 0.05, d = 0.22 [S]
	Significant others	18.05 (5.99)	18.99 (6.48)	*t*(842) = 5.29, *p* < 0.001, d = 0.37 [S]
	Total score	52.28 (14.63)	58.15 (13 .07)	*t*(842) = 5.99, *p* < 0.001, d = 0.42 [S]

Notes: AUD, amphetamine use disorder; HC, healthy controls; SD, standard deviation. [S] = small effect size; [L] = large effect size.

The groups of AUD and HC did not differ in gender distribution (χ^2^(N = 844, df = 1) = 1.37, *p* > 0.1) or age (*t*(842) = 1.23; *p* > 0.1, d = 0.23; see Table 2). Further, compared to HC, AUD were more likely to be married and divorced (χ^2^(N = 844, df = 2) = 36.35, *p* < 0.001), and they had higher educational degrees (χ^2^(N = 844, df = 3) = 48.75, *p* < 0.001). As regards the occupational status, compared to the statistical expectations, individuals with AUD were rather not students and homeworkers, and they were more unemployed and employed. Compared to the statistical expectations, individuals in the HC were rather students, and rather not unemployed, employed or homeworkers χ^2^(N = 844, df = 3) = 154.99, *p* < 0.001.

### 3.2 Symptoms of Insomnia and Depression, and Perceived Social Support in Participants with Amphetamine Use Disorder (AUD) and in Healthy Controls (HC)

Table 2 reports the descriptive and inferential statistical overview of age, symptoms of insomnia and depression and perceived social support in participants with AUD compared to HC.

Compared to HC, participants with AUD reported higher scores for insomnia (*t*(842) = 50,85, *p* < 0.001, d = 3.5 [L]) and depression (*t*(842) = 22.09, *p* < 0.001, d = 1.52 [L]). Further, participants with AUD reported lower scores for perceived social support (*t*(842) = 5.99, *p* < 0.001, d = 0.42 [S]), for social support from the family (*t*(842) = 5.90, *p* < 0.001, d = 0.41 [S]), from friends (*t*(842) = 3.11, *p* < 0.05, d = 0.22 [S]), and from significant others (*t*(842) = 5.29, *p* < 0.001, d = 0.37 [S]).

### 3.3 Associations between Symptoms of Insomnia and Depression, and Perceived Social Support (Full Sample of Participants with and without AUD)

Table 3 reports the Pearson’s correlations between symptoms of insomnia, depression and perceived social support.

**Table 3.** **Correlation coefficients between insomnia, depression and dimensions of social support**.

Dimensions		Insomnia	Depression	Social support	Social support	Social support	Social support
				Family	Friends	Significant others	Total score
Insomnia		-	0.58***	–0.18**	–0.16**	–0.16**	–0.21***
Depression			-	–0.26***	–0.09	–0.22**	–0.24***
Social support				-			
	Family				0.31***	0.62***	0.83***
	Friends				-	0.41***	0.68***
	Significant others					-	0.87***
	Total score						-

Notes: ** = *p* < 0.01; *** = *p* < 0.001.

The general pattern was as follows: Higher insomnia scores were associated with higher scores for depression, and lower scores for social support (family, friends, significant others, total score). Higher scores for depression were associated with lower scores for social support (family, friends, significant others, total score). Higher scores for social support (family, friends, significant others, total score) were inter-related.

### 3.4 Predictors to Identify Individuals with and without Amphetamine Use Disorder; Results from the Binary Regression Analysis

Table 4 reports the statistical indices of the precision and accuracy of the binary regression model to identify participants with AUD and HC based on their age, scores for insomnia and depression and perceived social support. Table 5 reports the statistical indices of the predictors.

**Table 4.** **Accuracy of correctly assign participants to the specific groups**.

	Predicted		Percentage correct
Observed	AUD	HC	
AUD	460	8	98.3
HC	14	361	96.3
Overall %			97.4

Notes: AUD, amphetamine use disorder; HC, healthy controls.

**Table 5.** **Binary regression model to correctly identify individuals in the AUD and HC condition**.

Dimension	B	S.E.	Wald	df	Sig.	Exp(B)
Constant	29.168	3397	73.720	1	<0.001	4.651 × 10^12^
Age	–0.172	0.042	16.350	1	<0.001	0.842
Gender	–0.253	0.464	0.298	1	0.585	0.776
Insomnia	–10.197	0.128	87.537	1	<0.001	0.302
Depression	–0.223	0.058	15.062	1	<0.001	0.800
Social support family	0.006	0.050	0.015	1	0.901	1.006
Social support friends	–0.048	0.051	0.904	1	0.342	0.953
Social support significant others	0.063	0.053	1.398	1	0.237	1.065

Notes: S.E., standard error.

The model summary is as follows:

–2 Log likelihood: 147.83; Cox & Snell R^2^: 0.698; Nagelkerke R^2^: 0.935, or simply put: The variance of the predictors explained 93.5% of the variance of the dependent variable.

The accuracy and precision of the binary regression analysis were 97.4%; 361 out of 375 HC (96.3% accuracy) were correctly assigned to the healthy control condition (14 out of 375 (3.7%) misleadingly assigned); 460 out of 468 individuals with AUD were correctly assigned to the group of individuals with AUD (98.3% accuracy; eight out of 468 (1.7%) were misleadingly assigned).

As shown in Table 5, the following dimensions identified the assignments correctly: Older age and higher scores for insomnia and depression, while gender and all dimensions of social support were excluded from the equation, as they did not reach statistical significance.

## 4. Discussion

The present study aimed to compare symptoms of insomnia and depression and dimensions of social support (family; friends; significant others) between individuals with amphetamine use disorders (AUD) and healthy controls (HC), and to identify which predictors were significant to correctly assign participants to their group. Data showed that compared to HC, individuals with AUD reported higher scores for insomnia and depression, and lower scores for social support. From the binary logistic regression model it turned out that higher age, and higher scores for depression and insomnia correctly assigned participants to the study condition with a precision of 97.4%, though, dimensions of social support were excluded from the equation, as they did not reach statistical significance. The present data add to the current scientific knowledge in the following three ways: First, to our understanding, the present data are novel in that we assessed adult Iranians with AUD. Second, from the comparison with healthy adults of the general population it turned out that individuals with AUD suffered also from symptoms of insomnia and depression, and decreased social support. Third, from the binary logistic model we identified age and symptoms of insomnia and depression as robust predictors to correctly assign participants to their specific groups (i.e., AUD vs. HC). The strength of the present study consists in a thorough assessment of the mental health status of adults with ‘pure’ AUD, this is to say: participants with AUD were carefully selected for not reporting further substance use issues and psychiatric issues.

Two hypotheses and one research question were formulated, and each of these is considered now in turn.

With the first hypothesis, we assumed that individuals with AUD would report higher scores for insomnia and depression than HC, and the present data did support this assumption and corroborate previous findings [16, 18, 30, 31, 32, 33, 34, 35, 36, 37]. However, the data expand upon previous findings in that we thoroughly assessed exclusively adults with AUD. Further, the degree of severity of AUD (i.e., following the DSM-5 [40] they met at maximum five criteria for AUD) was such that participants suffered from a mild to moderate AUD; they were not in a state to be hospitalized, but to follow their everyday life as much as possible.

With the second hypothesis, we expected that individuals with AUD would report lower scores for social support, compared to HC. As shown in Table 2, data did support these assumptions. As such, the present data confirmed and replicated previous research [19, 25, 26, 27, 28, 29]. However, a close inspection also revealed that effect sizes were small (ds between 0.22 and 0.42), or in other words: participants with AUD did not perceive their social environment (family, friends, significant others) as that much less supportive, compared to HC. The quality of the data does not allow a deeper understanding of that pattern of results. We speculate that despite their amphetamine-related issues, participants with AUD felt generally well embedded and supported by their social environment. It may also be possible that a sample bias occurred, in that we approached 712 individuals, though only about more than the half (n = 468; 53.84%) agreed to participate at the study. It is conceivable that compared to non-participants with AUD, participants with AUD were particularly motivated to report on their mental health status, and perhaps their overall impression was that their AUD was not impairing their everyday life so much. Last, though highly speculative, we might have assessed adults, who consumed amphetamine for non-medical reasons, and as such, perhaps they felt well accepted above all from their peers during their leisure time activities, such as attending gyms and clubs. Future studies in this field might assess participants’ motivation to use amphetamine regularly.

The research question was, whether and to what extent symptoms of depression, insomnia and social support from the family, peers and significant others, along with age and gender, would predict to assign individuals with AUD and healthy controls correctly to the specific study condition. The answer was that higher age and higher scores for depression and insomnia were the statistically only predictors, while dimensions of social support had to be excluded from the equation, as they did not reach statistical significance. In our opinion, this pattern of results matches well with what has been mentioned above as regards perceived social support: Considering the effect sizes (ds between 0.22 and 0.42 for all dimensions of perceived social support), participants with and without AUD did not substantively differ in the perceived social support. As such, the exclusion of social support as predictor in the binary regression model is coherent with the overall pattern of results.

Despite the novelty of the results, the following four limitations are considered: First, we fully relied on self-reports, and we did not perform any kind of biological toxicological screening (blood or urine samples) to rule out that participants used other substances. Importantly, this statement holds true for both individuals with AUD and HC. Second, by nature, cross-sectional studies do not allow causal relationships. More specifically, based on the cross-sectional nature of the present study, it remains unclear, whether symptoms of insomnia and depression, both of which are associated with cognitive impairments, encouraged a person to use amphetamines to counteract such cognitive deficits, or whether the use of amphetamines, which by nature impairs regular sleep patterns, caused both symptoms of insomnia and depression. By contrast, previous studies were able to identify a more complex and fine-grained bi-directional association between substance use and social support (see for instance [22, 24, 29]). However, we have run a binary logistic regression, which plausibly needs the definition of predictors and dependent variables. To do so, theoretical and previous data guided us to formulate the model. Third, the individual cognitive-emotional processes (e.g., [29]) and attachment styles (e.g., [48] for some suggestions) were not assessed. In our opinion, this would open a particularly important and fruitful research area, and the reasons are as follows: First, cognitive-emotional processes elaborate a person’s view of the world. More specifically, as regards social interactions, three basic processes are observed: (cf. [49, 50, 51, 52]). While an individual might react to constraints, rules and attitudes of a specific social context (reactive interaction), the pattern of personality also evokes distinctive responses from their social environment (evocative interaction)*.* Last, an individual also actively and selectively choses their social environment; or put the other way round: An individual is not only reactive and evocative to their social environment, but an individual also creates their social world (proactive interaction). Such proactive interactions may further shape and impact on the individual’s beliefs, how the social world appears to function (see also [29]). With this in mind, it would have been interesting to investigate, whether and to what extent dimensions of reactive, evocative and proactive behavior were associated with symptoms of depression, insomnia and social support among the present sample of individuals with AUD. More specifically, it would have been interesting to investigate, whether and to what extent their families, friends and significant others rather encouraged or discouraged the non-medical and recreational use of amphetamine. Fourth, we assessed a ‘pure’ sample of individuals with AUD, while the clinical experience shows that individuals consuming amphetamine often consume further substances such as cannabis, cocaine and opioids. In a similar vein, the co-occurrence of personality disorders or schizophrenia spectrum disorder is often observed. As such, it remains unclear, if and to what extent the present pattern of results is transferrable to a larger sample of individuals with AUD. Fifth, a possible confounding variable is the life history strategy [53, 54]. Briefly, the life history theory is a mid-level evolutionary framework to explain a person’s behaviors and outcomes such as mating strategies, risky behaviors, reproductive development, and health as a proxy of immediate resource provision. Typically, a person’s behavior is conceptualized as indicators of individual differences along a fast-slow life history continuum. To make the case in point, an individual adopting a fast strategy (that theoretically is most adaptive under harsh and unpredictable environmental conditions) uses short-term mating tactics, engages in risky behaviors, including the use of stimulants such as amphetamines, is less future oriented, and devotes less time to their offspring [53, 55, 56]. In a further consideration, and from an evolutionary perspective, use of stimulants may reflect personality indicators of a faster life strategy mirrored by lowered self-control, a short-term mating disposition, selfishness, and increased antisocial behavior [53, 57]. Given this, it appears highly conceivable that the assessment of fast vs. slow life history behavior might have helped to further understand the current pattern of amphetamine use. Sixth and last, research at the interface between psychiatry, biology and evolutionary psychology shows that individuals with opioid-, methamphetamine- [58], and amphetamine use disorder [54] had lower 2D:4D-ratios, and thus were most probably exposed prenatally to higher testosterone concentrations. Given this, it would have been interesting to measure participants’ finger lengths, too, to further investigate a possible biological factor for the tendency to use amphetamine.

## 5. Conclusions

Compared to healthy controls, adults with amphetamine use disorder reported additional mental health issues such as insomnia and depression, and lower perceived social support. Given that standardized psychotherapeutic interventions for insomnia, depression and social competencies are available, the implementation of such programs might also favorably impact on amphetamine use.

## Availability of Data and Materials

Data might be made available under the following conditions: (1) Whoever requests the data, is an expert in the field and senior researcher. (2) Requests are made via an institutional email-address (thus, no @gmail.com or @gmx.com or similar). (3) Hypotheses are clearly formulated. (4) The expert in the field and the institution confirm and guarantee, that all data are securely stored on a server of the institution, where nobody else has access to. (5) The expert in the field and the institution confirm and guarantee, that data won’t be shared with third parties, even when the third party belongs to the same institution, department or research team.

## References

[1] Stone AL, Becker LG, Huber AM, Catalano RF (2012). Review of risk and protective factors of substance use and problem use in emerging adulthood. *Addictive Behaviors*.

[2] United Nations Office of Drugs and Crime (2023). *World Drug Report 2023*.

[3] Faraone SV, Asherson P, Banaschewski T, Biederman J, Buitelaar JK, Ramos-Quiroga JA (2015). Attention-deficit/hyperactivity disorder. *Nature Reviews. Disease Primers*.

[4] Cortese S (2020). Pharmacologic Treatment of Attention Deficit-Hyperactivity Disorder. *The New England Journal of Medicine*.

[5] Groom MJ, Cortese S (2022). Current Pharmacological Treatments for ADHD. *Current Topics in Behavioral Neurosciences*.

[6] Bassetti CLA, Kallweit U, Vignatelli L, Plazzi G, Lecendreux M, Baldin E (2021). European guideline and expert statements on the management of narcolepsy in adults and children. *Journal of Sleep Research*.

[7] Heal DJ, Smith SL, Gosden J, Nutt DJ (2013). Amphetamine, past and present–a pharmacological and clinical perspective. *Journal of Psychopharmacology*.

[8] Liddle DG, Connor DJ (2013). Nutritional supplements and ergogenic AIDS. *Primary Care*.

[9] Vitiello B (2008). Understanding the risk of using medications for attention deficit hyperactivity disorder with respect to physical growth and cardiovascular function. *Child and Adolescent Psychiatric Clinics of North America*.

[10] Stahl SM (2017). *Prescriber’s Guide: Stahl’s Essential Psychopharmacology*.

[11] Benz F, Meneo D, Baglioni C, Hertenstein E (2023). Insomnia symptoms as risk factor for somatic disorders: An umbrella review of systematic reviews and meta-analyses. *Journal of Sleep Research*.

[12] Hertenstein E, Benz F, Schneider CL, Baglioni C (2023). Insomnia-A risk factor for mental disorders. *Journal of Sleep Research*.

[13] Hertenstein E, Feige B, Gmeiner T, Kienzler C, Spiegelhalder K, Johann A (2019). Insomnia as a predictor of mental disorders: A systematic review and meta-analysis. *Sleep Medicine Reviews*.

[14] Palagini L, Hertenstein E, Riemann D, Nissen C (2022). Sleep, insomnia and mental health. *Journal of Sleep Research*.

[15] Boland EM, Goldschmied JR, Gehrman PR (2023). Does insomnia treatment prevent depression?. *Sleep*.

[16] Crouse JJ, Lee RSC, White D, Moustafa AA, Hickie IB, Hermens DF (2018). Distress and sleep quality in young amphetamine-type stimulant users with an affective or psychotic illness. *Psychiatry Research*.

[17] Regmi S, Kedia SK, Schmidt M, Mahmood A, Lugemwa T, Dillon PJ (2024). Methamphetamine-Induced Wakefulness and Sleep Management: A Qualitative Analysis of Online Narratives. *Journal of Psychoactive Drugs*.

[18] Leung J, Mekonen T, Wang X, Arunogiri S, Degenhardt L, McKetin R (2023). Methamphetamine exposure and depression-A systematic review and meta-analysis. *Drug and Alcohol Review*.

[19] Bonar EE, Walton MA, Carter PM, Lin LA, Coughlin LN, Goldstick JE (2022). Longitudinal within- and between-person associations of substance use, social influences, and loneliness among adolescents and emerging adults who use drugs. *Addiction Research & Theory*.

[20] Ingram I, Kelly PJ, Deane FP, Baker AL, Goh MCW, Raftery DK (2020). Loneliness among people with substance use problems: A narrative systematic review. *Drug and Alcohol Review*.

[21] Shahid A, Asmat A (2023). Stigmatisation and perceived social support as predictor of treatment of substance use disorder (SUD): A systematic review. *JPMA. the Journal of the Pakistan Medical Association*.

[22] Armoon B, Fleury MJ, Bayat AH, Bayani A, Mohammadi R, Griffiths MD (2022). Quality of life and its correlated factors among patients with substance use disorders: a systematic review and meta-analysis. *Archives of Public Health*.

[23] Birkeland B, Weimand B, Ruud T, Maybery D, Vederhus JK (2021). Perceived family cohesion, social support, and quality of life in patients undergoing treatment for substance use disorders compared with patients with mental and physical disorders. *Addiction Science & Clinical Practice*.

[24] Rathinam B, Ezhumalai S (2022). Perceived Social Support among Abstinent Individuals with Substance use disorder. *Journal of Psychosocial Rehabilitation and Mental Health*.

[25] Georgie J M, Sean H, Deborah M C, Matthew H, Rona C (2016). Peer-led interventions to prevent tobacco, alcohol and/or drug use among young people aged 11-21 years: a systematic review and meta-analysis. *Addiction*.

[26] Hunter RF, de la Haye K, Murray JM, Badham J, Valente TW, Clarke M (2019). Social network interventions for health behaviours and outcomes: A systematic review and meta-analysis. *PLoS Medicine*.

[27] Stevens E, Jason LA, Ram D, Light J (2015). Investigating Social Support and Network Relationships in Substance Use Disorder Recovery. *Substance Abuse*.

[28] Swinkels LTA, Hoeve M, Ter Harmsel JF, Schoonmade LJ, Dekker JJM, Popma A (2023). The effectiveness of social network interventions for psychiatric patients: A systematic review and meta-analysis. *Clinical Psychology Review*.

[29] Winters DE, Brandon-Friedman R, Yepes G, Hinckley JD (2021). Systematic review and meta-analysis of socio-cognitive and socio-affective processes association with adolescent substance use. *Drug and Alcohol Dependence*.

[30] Bhagavan C, Kung S, Doppen M, John M, Vakalalabure I, Oldfield K (2020). Cannabinoids in the Treatment of Insomnia Disorder: A Systematic Review and Meta-Analysis. *CNS Drugs*.

[31] Amaral C, Carvalho C, Scaranelo A, Chapman K, Chatkin J, Ferreira I (2023). Cannabis and sleep disorders: not ready for prime time? A qualitative scoping review. *Journal of Clinical Sleep Medicine*.

[32] Gonzalez JE, Shea SA, Bowles NP (2023). Daily cannabis use is associated with sleep duration differentially across ages. *Sleep Health*.

[33] Graupensperger S, Fairlie AM, Ramirez JJ, Calhoun BH, Patrick ME, Lee CM (2022). Daily-level associations between sleep duration and next-day alcohol and cannabis craving and use in young adults. *Addictive Behaviors*.

[34] Diep C, Tian C, Vachhani K, Won C, Wijeysundera DN, Clarke H (2022). Recent cannabis use and nightly sleep duration in adults: a population analysis of the NHANES from 2005 to 2018. *Regional Anesthesia and Pain Medicine*.

[35] Gobbi G, Atkin T, Zytynski T, Wang S, Askari S, Boruff J (2019). Association of Cannabis Use in Adolescence and Risk of Depression, Anxiety, and Suicidality in Young Adulthood: A Systematic Review and Meta-analysis. *JAMA Psychiatry*.

[36] Onaemo VN, Fawehinmi TO, D’Arcy C (2021). Comorbid Cannabis Use Disorder with Major Depression and Generalized Anxiety Disorder: A Systematic Review with Meta-analysis of Nationally Representative Epidemiological Surveys. *Journal of Affective Disorders*.

[37] Hunt GE, Malhi GS, Lai HMX, Cleary M (2020). Prevalence of comorbid substance use in major depressive disorder in community and clinical settings, 1990-2019: Systematic review and meta-analysis. *Journal of Affective Disorders*.

[38] World Medical Association (2013). World Medical Association Declaration of Helsinki: ethical principles for medical research involving human subjects. *JAMA*.

[39] First M (2015). *Structured Clinical Interview for the DSM (SCID). The Encyclopedia of Clinical Psychology*.

[40] American Psychiatric Association (2013). *Diagnostic and Statistical Manual of Mental Disorders*.

[41] Haidari R, Najafi A, Jahanbakhsh F, Efanian R, Yazdani M (2022). Validity and reliability of the Persian version of the Athens Insomnia Scale. *ASEAN Journal of Psychiatry*.

[42] Soldatos CR, Dikeos DG, Paparrigopoulos TJ (2000). Athens Insomnia Scale: validation of an instrument based on ICD-10 criteria. *Journal of Psychosomatic Research*.

[43] Habibi M, Hamediniya E, Asgarinejad F, Kholghi H (2016). Psychometric Properties of Kutcher Adole Csent’s Depression Scale. *AEP*.

[44] Brooks SJ, Kutcher S (2001). Diagnosis and measurement of adolescent depression: a review of commonly utilized instruments. *Journal of Child and Adolescent Psychopharmacology*.

[45] Mousavi A, Shojaee M, Shahidi M, Cui Y, Kutcher S (2019). Measurement invariance and psychometric analysis of Kutcher Adolescent Depression Scale across gender and marital status. *Journal of Affective Disorders*.

[46] Bagherian-Sararoudi R, Hajian A, Ehsan HB, Sarafraz MR, Zimet GD (2013). Psychometric properties of the persian version of the multidimensional scale of perceived social support in iran. *International Journal of Preventive Medicine*.

[47] Zimet GD, Powell SS, Farley GK, Werkman S, Berkoff KA (1990). Psychometric characteristics of the Multidimensional Scale of Perceived Social Support. *Journal of Personality Assessment*.

[48] Golshani S, Najafpour A, Hashemian SS, Goudarzi N, Firoozabadi A, Ghezelbash MS (2021). Individuals with Major Depressive Disorder Report High Scores of Insecure-Avoidant and Insecure-Anxious Attachment Styles, Dissociative Identity Symptoms, and Adult Traumatic Events. *Healthcare*.

[49] Bouchard Jr TJ (2004). Genetic Influence on Human Psychological Traits: A Survey. *Current Directions in Psychological Science*.

[50] Scarr S (1992). Developmental theories for the 1990s: development and individual differences. *Child Development*.

[51] Scarr S (1996). How people make their own environments: Implications for parents and policy makers. *Psychology, Public Policy, and Law*.

[52] Scarr S, McCartney K (1983). How people make their own environments: a theory of genotype greater than environment effects. *Child Development*.

[53] Marchegiani V, Zampieri F, Della Barbera M, Troisi A (2018). Gender differences in the interrelations between digit ratio, psychopathic traits and life history strategies. *Personality and Individual Differences*.

[54] Hashemian SS, Golshani S, Firoozabadi K, Firoozabadi A, Fichter C, Dürsteler KM (2024). 2D:4D-ratios among individuals with amphetamine use disorder, antisocial personality disorder and with both amphetamine use disorder and antisocial personality disorder. *Journal of Psychiatric Research*.

[55] Chua KJ, Lukaszewski AW, Grant DM, Sng O (2017). Human Life History Strategies. *Evolutionary Psychology: an International Journal of Evolutionary Approaches to Psychology and Behavior*.

[56] Strouts PH, Brase GL, Dillon HM (2017). Personality and evolutionary strategies: The relationships between HEXACO traits, mate value, life history strategy, and sociosexuality. *Personality and Individual Differences*.

[57] Jonason PK, Koenig BL, Tost J (2010). Living a Fast Life. *Human Nature*.

[58] Akkuş M, Avşar PA (2024). 2D:4D ratio, alexithymia, impulsivity, aggression, and ADHD in men with opioid and methamphetamine use disorders: A comparative analysis with healthy controls. *Early Human Development*.

